# Benefits of spatial uncertainty aggregation for segmentation in digital pathology

**DOI:** 10.1117/1.JMI.11.1.017501

**Published:** 2024-01-16

**Authors:** Milda Pocevičiūtė, Gabriel Eilertsen, Claes Lundström

**Affiliations:** a Linköping University, Center for Medical Image Science and Visualization, Linköping, Sweden; b Linköping University, Department of Science and Technology, Linköping, Sweden; c Sectra AB, Linköping, Sweden

**Keywords:** computational pathology, deep learning, uncertainty estimation, false negative detection, tumor metastases segmentation

## Abstract

**Purpose:**

Uncertainty estimation has gained significant attention in recent years for its potential to enhance the performance of deep learning (DL) algorithms in medical applications and even potentially address domain shift challenges. However, it is not straightforward to incorporate uncertainty estimation with a DL system to achieve a tangible positive effect. The objective of our work is to evaluate if the proposed spatial uncertainty aggregation (SUA) framework may improve the effectiveness of uncertainty estimation in segmentation tasks. We evaluate if SUA boosts the observed correlation between the uncertainty estimates and false negative (FN) predictions. We also investigate if the observed benefits can translate to tangible improvements in segmentation performance.

**Approach:**

Our SUA framework processes negative prediction regions from a segmentation algorithm and detects FNs based on an aggregated uncertainty score. It can be utilized with many existing uncertainty estimation methods to boost their performance. We compare the SUA framework with a baseline of processing individual pixel’s uncertainty independently.

**Results:**

The results demonstrate that SUA is able to detect FN regions. It achieved $F_{\beta =0.5}$ of 0.92 on the in-domain and 0.85 on the domain-shift test data compared with 0.81 and 0.48 achieved by the baseline uncertainty, respectively. We also demonstrate that SUA yields improved general segmentation performance compared with utilizing the baseline uncertainty.

**Conclusions:**

We propose the SUA framework for incorporating and utilizing uncertainty estimates for FN detection in DL segmentation algorithms for histopathology. The evaluation confirms the benefits of our approach compared with assessing pixel uncertainty independently.

## 1 Introduction

The utilization of deep learning (DL) in pathology departments has the potential to significantly improve patient care by supporting physicians with tasks such as providing melanoma diagnosis,^1^ detecting breast cancer metastases,^2^^–^^4^ and grading prostate cancer.^5^^–^^8^ Nevertheless, DL systems for medical imaging are known to encounter generalization issues due to their sensitivity to outliers and domain shift, i.e., a change in the underlying data distribution.^9^^,^^10^ This can be encountered when an algorithm is deployed in a new medical center as well as in the same medical center over time.^11^ Relying solely on the DL output score to assess the reliability of the predictions often does not work well due to the commonly observed over-confidence of such algorithms.^12^ Therefore, developing and deploying new methods for assessing the dependability of the DL predictions is essential for wider clinical acceptance of the technology.^13^ Uncertainty estimation has been proposed as one of the solutions that could potentially improve the performance and robustness of DL systems.^14^ However, deriving the maximum benefit of uncertainty estimation in segmentation DL for digital pathology is a complex task requiring careful evaluations.

This work focuses on DL application to breast cancer metastasis segmentation as this type of cancer is one of the most prevalent cancers worldwide.^15^ To provide appropriate treatment, it is crucial to determine if cancerous cells have spread to other organs and tissues. Typically, the nearest lymph nodes are removed surgically, and tissue samples are fixed, sliced, and stained with hematoxylin and eosin (H & E) to create glass slides. This procedure is referred to as sentinel lymph node dissection. A pathologist then carefully examines the glass slides or digitized whole slide images (WSIs) to detect potential tumor metastases. This can be a labor-intensive and time-consuming process, particularly because multiple WSIs from each patient may require examination.^2^ Therefore, assistance from DL systems could prove to be valuable.^16^^–^^18^

We propose a spatial uncertainty aggregation (SUA) framework for improving the effectiveness of uncertainty estimation of a DL model trained to segment breast cancer metastases in lymph nodes. We hypothesize that the overall confidence of a neighborhood of pixels may contain additional information; hence we aggregate segmentation predictions into regions and compute an uncertainty measure for each of them. Our results show that utilizing spatial information works better than considering pixels independently.

We focus on clinical relevance; hence in the evaluation of the proposed method we (a) fix the segmentation threshold instead of relying on threshold-independent metrics and (b) work on false negatives (FN) detection as this task often requires more time from pathologists than false positive (FP) rejection. Importantly, the SUA framework is agnostic to the uncertainty estimation technique and requires no intervention from pathologists. In the study, we utilize the deep ensemble (DE) uncertainty estimation method^19^ due to the promising results exhibited in related work.^14^^,^^20^ Our experiments reveal a strong correlation between aggregated uncertainty and incorrect segmentation, and we explore whether this information can enhance the performance of a DL diagnostic system.

## 2 Related Work

Several techniques have been developed to estimate uncertainty in DL, such as test time augmentations,^21^ Monte Carlo dropout,^22^ and DEs.^19^ Previous studies have demonstrated the usefulness of estimating uncertainty in computational pathology, in which a common strategy involves identifying the most uncertain predictions for manual review by medical professionals, allowing them to focus on challenging cases.^23^^,^^24^ Similarly, uncertainty heatmaps can be generated and superimposed on the original image for visual inspection.^25^^,^^26^ The latter approach can be valuable during the algorithm development phase, but it is impractical for pathologists under time constraints in clinical production.

Alternatively, incorporating uncertainty estimates into the DL framework may not require intervention from a physician. For example, previous works have examined the correlation between estimated uncertainty and mispredictions^14^^,^^20^ or outliers^27^ in classification, which could be used to reduce the error in performance. In addition, combining uncertainty with the softmax output may enhance the generalizability and robustness of DL-based classifiers for histopathology applications.^14^ In segmentation, it has been shown that uncertainty can be used to filter out FP prediction areas on digital pathology data.^28^ Our work falls under this direction of research as it is focused on determining how uncertainty estimation could be used to refine the segmentation predictions by detecting FNs.

## 3 Method

### 3.1 Slide Heatmaps

The proposed SUA framework (see Sec. 3.3) involves, apart from the original WSI, two computed scalar valued maps for each slide. The first is the pixel-wise softmax output from the segmentation NN, which we refer to as the segmentation heatmap. The second map is the output from the uncertainty estimation step with pixel-wise uncertainty values, referred to as the uncertainty heatmap.

### 3.2 Negative Prediction Regions

The SUA framework targets the detection of FN regions. The analysis is performed for what we denote as negative prediction regions (NPRs). An NPR is defined as a cluster of adjacent pixels that have been classified as negative by the DL algorithm but are reasonable candidates for being FN pixels. Notably, NPRs do not need to be adjacent to the areas that are already predicted positive by the DL algorithm. NPRs are determined by locating groups of adjacent pixels that are assigned a softmax value output by the DL model within a chosen interval. The upper threshold is the cutoff value for a positive prediction. The lower softmax bound is used to avoid excessively large NPRs. Algorithm 1 provides a pseudo code for determining NPRs in a WSI. The function for labeling the connected regions, i.e., the skimage.measure.label function from the sklearn library,^29^ is based on the work of Wu et al.^30^ We explored the impact of different settings of the lower threshold: 0.55, 0.65, 0.75, and 0.85.

**Algorithm 1:** Determining NPRs in a given WSI.

**Input:** segmentation Heatmap, lowerThreshold, upperThreshold
t1←lowerThreshold
t2←upperThreshold
binarySegmentation=(segmentationHeatmap>t1)&(segmentationHeatmap<t2)
nprIslands=skimage.measure.label(binarySegmentation,connectivity=2)
**Output:** nprIslands

**Fig. 1:**
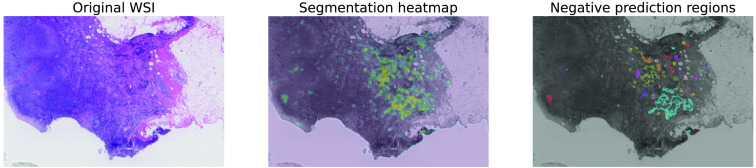
Visualization of NPRs. The leftmost image shows a part of the original WSI, and the middle one shows the segmentation heatmap produced by a DL model. In the rightmost image, NPRs are visualized in different colors. They are determined by finding adjacent pixels that are assigned a segmentation softmax score in a predetermined range (0.55 to 0.95 in this example).

The segmentation threshold is set to 0.95 in our study as this results in eight average FPs per WSIs on the validation data. This decision was based on the free-response receiver operating characteristic (FROC) metric,^2^ which assesses the clinical relevance of metastases detection algorithms. Therefore, the evaluated NPRs were built considering pixels with softmax scores in the ranges of 0.55 to 0.95, 0.65 to 0.95, 0.75 to 0.95, and 0.85 to 0.95. We also experimented with smaller NPRs, i.e., with softmax scores in the 0.55 to 0.65 and 0.65 to 0.75 ranges, but both SUA and the baseline (see Sec. 3.4) showed substantially inferior performance; hence we excluded them from the results. Figure 1 visualizes an example of a WSI with its NPRs.

### 3.3 Spatial Uncertainty Aggregation framework

**Fig. 2:**
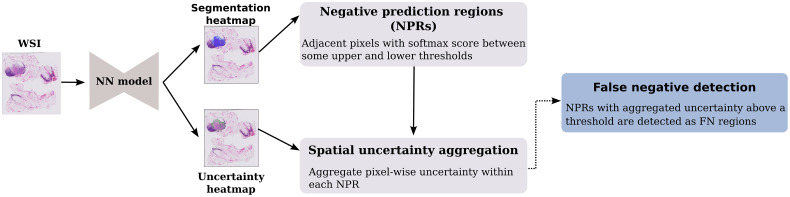
Outline of the SUA framework that utilizes spatially aggregated uncertainty to identify FNs.

Figure 2 shows the SUA framework. Given a segmentation algorithm and uncertainty estimation method, segmentation and uncertainty heatmaps are generated. As discussed above, two thresholds are employed to create NPRs: the upper threshold identifies pixels predicted to be of a positive class, whereas the lower threshold is utilized to restrict the NPRs’ areas. Within NPRs, the uncertainty values are aggregated and applied to distinguish between true negative (TN) and FN predictions. We evaluate two different aggregation functions: the average and the 90th percentile of the values. Note that any uncertainty estimation technique can be utilized in the SUA framework. If the resulting aggregated uncertainty score is above a set threshold, the NPR is marked as an FN region. This threshold is empirically determined on validation data.

### 3.4 Baseline

To evaluate if we benefit from having the spatial information in the proposed SUA framework, we compare its performance with a baseline in which all pixels and their corresponding entropy scores are treated independently, that is, the baseline is the direct utilization of the underlying uncertainty method. It is computed for the same pixels as those in NPRs used by the proposed SUA method.

## 4 Implementation Details and Data

### 4.1 Segmentation NN

The segmentation neural networks (NNs) used in this study were built upon the DenseNet architecture^31^ and closely followed the implementation in previous work.^18^^,^^32^ During training, the ADAM optimizer with $\beta_{1}=0.9$ and $\beta_{2}=0.999$ was utilized, and the initial learning rate was set to 0.01. In case there was no improvement on the validation data for 20 consecutive epochs, a learning rate decay of 0.1 was applied. To reflect the clinical situation in which negative slides are much more common than positive ones, the training process used four times more healthy slides than tumor patches.^18^^,^^32^ In addition, the training augmentations closely followed the recommendations by Tellez et al.^9^ Each model was initialized with the He initialization scheme^33^ using a different random seed, and the maximum training limit was set to 250 epochs but stopped earlier if convergence was observed. To segment tumor areas in lymph node WSIs, we trained five segmentation NNs with different random seeds and averaged their softmax score predictions per pixel. We needed five NNs to estimate the uncertainty of the prediction; see the next section.

### 4.2 Uncertainty Estimation

In our experiments, the DEs uncertainty quantification method^19^ is used to estimate the epistemic uncertainty for each pixel. This requires an ensemble of identical NNs with different random seeds. We used an ensemble of five NNs. To quantify the uncertainty from the multiple predictions, we adopted an entropy measure.^34^ Specifically, for a random variable X with possible outcomes $x_{i},i\in \{0,1,..\}$, the entropy is defined as follows:

$$H(X)=-\underset{i}{\sum }P(x_{i})\log P(x_{i}),$$

which we approximate as proposed by Gal et al,^35^ that is, $x_{i}$ in our case represents an average softmax prediction by the ensemble for class i and the entropy is computed for each pixel independently.

In addition to the ensemble setup described above, we tried several other alternatives to explore whether other types of variability would improve the uncertainty estimation. We created and evaluated several ensembles consisting of up to 13 NNs. The variation in the NNs was introduced by different training strategies such as sampling different proportions of tumor versus healthy patches and selecting different optimizers, i.e., RMSProp, Nadam, and SGD. However, we observed no significant difference in the performance on FN detection. Therefore, we only present the results acquired by utilizing the five identical segmentation NNs trained with different random seeds as described in Sec. 4.1.

### 4.3 Data

A total of 344 WSIs from the Camelyon Grand Challenge (CGC) dataset^36^ were utilized for segmentation model development. Specifically, 271 WSIs (104 WSIs with tumor metastasis) were used for training, and 73 WSIs (31 WSIs with tumor metastasis) were used for validation. The split was done on the patient level. In addition, a set of 139 WSIs with 59 WSIs containing tumor metastases was reserved for in-domain testing, which is referred to as the Camelyon data in this study. The total number of WSIs taken from the CGC dataset was limited by the availability of detailed annotations. For testing the model’s performance on domain-shift data, a subset of 164 WSIs (57 WSIs with tumor metastases) from the AIDA BRLN dataset^37^ was selected. In the following sections, we refer to this dataset as the Sentinel data. All used datasets are publicly available to be utilized in legal and ethical medical diagnostics research.

## 5 Experiments

We conducted two experiments that are briefly described below. The goal was to evaluate the potential of the SUA framework to enhance the usefulness of the underlying uncertainty estimates and investigate its practical impact on boosting the performance of segmentation NNs in in-domain and domain-shift scenarios.

### 5.1 Experiment 1: Correlation Analysis

The primary objective of the first experiment is to determine whether there is a basis to assume that spatially aggregated uncertainty improves the usefulness of uncertainty information. This goal is accomplished by examining the relationship between the FN segmentation regions and the spatially aggregated uncertainty, i.e., how well uncertainty correlates with incorrect predictions. Initial investigations indicate that the vast majority of NPRs with at least one pixel incorrectly classified to be negative were comprised of more than 90% of misclassified pixels. Consequently, we define FNs as NPRs with at least 90% missed tumor pixels. This means that detecting these FN NPRs would not introduce many FP pixel predictions. The pixel-wise correlation between uncertainty and FN predictions provided by the baseline method is reported for comparison.

### 5.2 Experiment 2: Segmentation Heatmaps Refinement

The second experiment aims to assess the impact of the proposed SUA framework on the segmentation performance of a DL system. This is accomplished by utilizing SUA FN detection for refinement of segmentation heatmaps. If the spatial uncertainty value of an NPR exceeds a pre-defined detection threshold, the prediction for that region is updated to be positive. This is compared with the outcome of refining the prediction heatmaps using the independent pixel-wise uncertainty of the baseline method. In this case, the softmax score of a pixel is updated if the uncertainty of that pixel is above a pre-defined detection threshold. This is done without any consideration to the other pixels that belong to the same NPR. In both cases, the optimal detection threshold is determined on the validation data.

### 5.3 Evaluation

In experiment 1, $F_{\beta =0.5}$ score is used to evaluate the ability to distinguish between FN and TN regions. It is defined as^38^

$$F_{\beta }=(1+\beta^{2})\frac{\mathrm{precision}\cdot \mathrm{recall}}{(\beta^{2}\cdot \mathrm{precision})+\mathrm{recall}}.$$

We set β=0.5, which weights precision more than recall because having a high precision reduces the risk of introducing a large amount of falsely segmented pixels.

In experiment 2, we do a threefold assessment of potential practical gains from utilizing the FN detection for WSI segmentation refinement. First, the average Dice score over all WSIs in a given dataset is compared before and after the refinement of the segmentation heatmaps. Given the number of true positive (TP), FP, and FN pixel predictions, the Dice score is defined as^39^

$$\mathrm{DICE}=\frac{2\cdot \mathrm{TP}}{2\cdot \mathrm{TP}+\mathrm{FP}+\mathrm{FN}}.$$

Due to the vast number of tumor pixels in WSIs and, in comparison, the small number of pixels with updated predictions, the average Dice score alone may be incapable of revealing the full impact of the refinement.^38^ To address this issue and enable a better comparison between the baseline and SUA methods, we propose a metric called the false negative conversion rate (FNCR). It quantifies what proportion of the updated negative-prediction pixels actually belong to the positive class, that is, given a dataset $A_{K}=\{{\mathrm{WSI}}_{1},\ldots ,{\mathrm{WSI}}_{K}\}$ and the corresponding refined segmentation heatmaps via the SUA framework or the baseline, the FNCR is defined as

$$\mathrm{FNCR}=\frac{\overset{K}{\underset{i=1}{\sum }}N_{+}^{i}}{\overset{K}{\underset{i=1}{\sum }}N_{\mathrm{total}}^{i}},$$

where $N_{+}^{i}$ is the number of pixels that were originally considered negative and correctly updated to be positive, i.e., detected FNs, and $N_{\mathrm{total}}^{i}$ is the total number of pixels updated in some ${\mathrm{WSI}}_{i}\in A_{K}$. If the FNCR is 1, it means that only FN pixels were updated, whereas having an FNCR close to 0 indicates that the refinement mostly introduced FP predictions.

We also report the observed change in FROC-AUC^2^ and ROC-AUC^39^ after the refinement, but we deem this to be the least informative approach. As our aim is to improve lesion segmentation within a slide, this should have a minimal effect on ROC-AUC, which quantifies the performance of classifying the WSIs. The problem with the FROC-AUC metric is that it determines unique optimal thresholds for each dataset. The thresholds that we fixed based on the validation data may be substantially different from the thresholds employed in the FROC-AUC computation on the test sets. We argue that, in a clinical setting, an operating threshold needs to be fixed and, hence, continuously adapting it to the incoming new data usually is unfeasible.

## 6 Results

### 6.1 Segmentation Performance

Table 1 shows that the ensemble achieved close to the state-of-the-art performance on the primary task of breast cancer metastasis detection. The best reported result on the Camelyon16 Grand Challenge is 0.81 FROC-AUC and 0.99 ROC-AUC;^40^ however, our test data included images from both the Camelyon16 and the Camelyon17 datasets. As expected, the performance dropped on the domain-shift Sentinel data with a relatively large difference in the FROC-AUC value and a smaller change in the ROC-AUC value and average DICE score.

**Table 1:** Lymph node metastasis in breast cancer segmentation results reported in ROC-AUC, FROC-AUC, and average Dice scores. The mean and standard deviation (in brackets) values are computed over the 1000 bootstrapped iterations.

Dataset	FROC-AUC	ROC-AUC	Dice score
Camelyon	0.83 (0.05)	0.97 (0.02)	0.71 (0.02)
Sentinel	0.68 (0.10)	0.96 (0.02)	0.70 (0.03)

### 6.2 Experiment 1

**Fig. 3:**
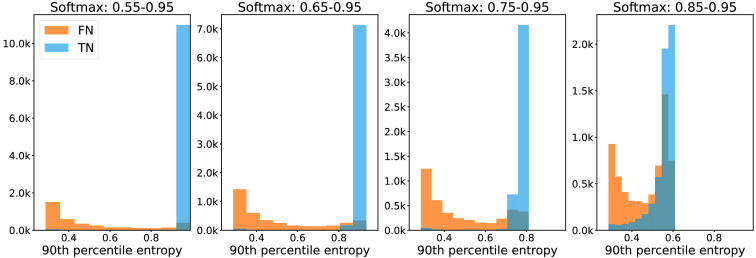
Histogram of the 90th percentile entropy of the NPRs built using varying softmax ranges. FNs are the islands that have at least 90% overlap with the ground truth tumor annotation. Camelyon validation data.

**Fig. 4:**
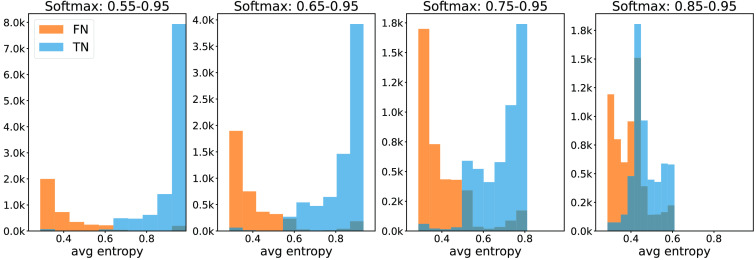
Histogram of the average entropy of the NPRs built using varying softmax ranges. FNs are the islands that have at least 90% overlap with the ground truth tumor annotation. Camelyon validation data.

**Fig. 5:**
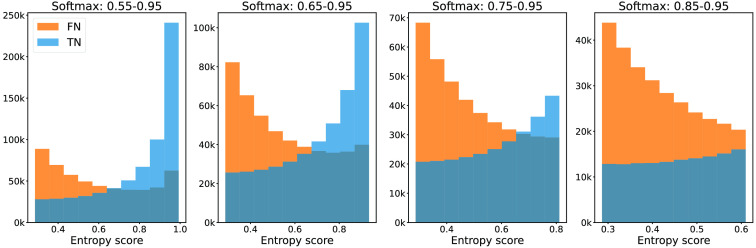
Histogram of the baseline pixel entropy divided between TN and FN predictions. Pixels analyzed with softmax values in the ranges of 0.55 to 0.95, 0.65 to 0.95, 0.75 to 0.95, and 0.85 to 0.95. Camelyon validation data.

From the histograms in Figs. 3 and 4, we conclude that the separation between FNs and TNs achieved by the 90th percentile and the average uncertainty aggregation approaches are similarly good. $F_{\beta =0.5}$ scores computed on the validation data revealed that using average entropy resulted in a marginally better performance. Hence, we report the results using the average uncertainty aggregation approach in the remainder of the work. Figure 5 shows a corresponding histogram using the baseline method, i.e., processing pixels entropy without spatial aggregation. Less separation between FN and TN predictions for the considered softmax score ranges is achieved in this case.

Table 2 shows the $F_{\beta =0.5}$ scores achieved by the SUA framework with different sizes of NPRs and the baseline with a matching constraint on the softmax score of considered pixels. It confirms the observation that aggregating uncertainty spatially results in a better separation between TN and FN predictions in almost all considered scenarios. Overall, the highest mean $F_{\beta =0.5}$ scores on the Camelyon and Sentinel datasets of 0.92 and 0.86, respectively, were achieved by the SUA framework.

**Table 2:** $F_{\beta =0.5}$ performance of FN versus TN differentiation on the Camelyon and Sentinel test datasets. In the SUA method, an NPR is considered to be FN if it contains at least 90% tumor pixels. The mean and standard deviation values are computed over the 1000 bootstrapped iterations.

Dataset	Method	$F_{\beta =0.5}$ with softmax scores in ranges			
		0.55 to 0.95	0.65 to 0.95	0.75 to 0.95	0.85 to 0.95
Camelyon	Baseline	0.71 (0.05)	0.74 (0.06)	0.76 (0.06)	0.81 (0.06)
	SUA	0.92 (0.02)	0.91 (0.02)	0.89 (0.02)	0.73 (0.04)
Sentinel	Baseline	0.42 (0.06)	0.43 (0.06)	0.45 (0.06)	0.48 (0.06)
	SUA	0.86 (0.04)	0.85 (0.04)	0.83 (0.04)	0.64 (0.05)

### 6.3 Experiment 2

Table 3 summarizes how the average Dice score is affected by applying SUA and the baseline for refinement of the segmentation heatmaps. We can see that updated heatmaps through the baseline method systematically result in lower average Dice scores than the original predictions. The SUA framework achieves higher average DICE scores than observed on the original predictions when NPRs were computed using 0.55 to 0.95 and 0.65 to 0.95 softmax ranges on both test sets. Figure 7 shows an example of a segmentation refined by applying the SUA framework.

**Table 3:** Average Dice score before and after segmentation refinement by the SUA and baseline methods. The results are reported per considered softmax ranges, i.e., 0.55 to 0.95, 0.65 to 0.95, 0.75 to 0.95, and 0.85 to 0.95. The mean and standard deviation values are computed over the 1000 bootstrapped samples.

Dataset	Method	Original Dice	Dice scores after update, softmax in ranges			
			0.55 to 0.95	0.65 to 0.95	0.75 to 0.95	0.85 to 0.95
Camelyon	Baseline	0.706 (0.023)	0.676	0.628	0.654	0.680
			(0.025)	(0.028)	(0.026)	(0.024)
	SUA		0.709	0.709	0.706	0.700
			(0.024)	(0.024)	(0.024)	(0.024)
Sentinel	Baseline	0.700 (0.026)	0.672	0.672	0.676	0.681
			(0.028)	(0.028)	(0.027)	(0.027)
	SUA		0.704	0.703	0.702	0.695
			(0.026)	(0.026)	(0.026)	(0.026)

**Fig. 6:**
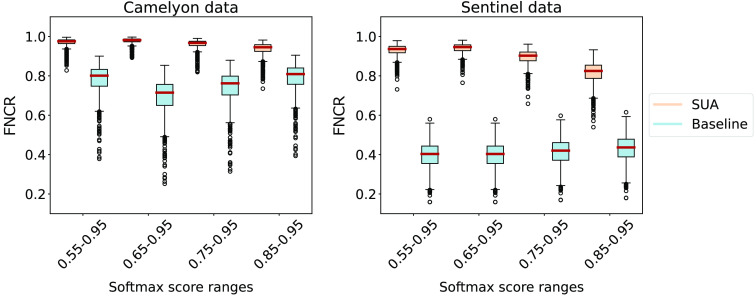
FNCR achieved by SUA and the baseline on the Camelyon and Sentinel datasets with 1000 bootstrap iterations. The red horizontal line in each box indicates the median value. The results are reported per considered softmax ranges, i.e., 0.55 to 0.95, 0.65 to 0.95, 0.75 to 0.95, and 0.85 to 0.95.

The box plots in Fig. 6 summarize the results of the FNCR metric computed over 1000 bootstrap iterations. SUA achieved substantially higher median values in all scenarios compared with the baseline. Domain shift has an impact on SUA effectiveness as the median values fall from being between 0.98 and 0.95 on the Camelyon data to 0.94 and 0.82 on the Sentinel data for the considered NPRs. However, the effectiveness of the baseline in FN detection suffers a much sharper drop due to the domain shift: from around 0.8 to around 0.4 on the Camelyon and Sentinel data, respectively.

Table 4 shows the number of negative WSIs that had some FN areas introduced after the refinement using the SUA and baseline methods. SUA incorrectly updated significantly fewer such WSIs in all considered scenarios. In fact, the baseline method refined nearly all negative WSIs in the Camelyon and Sentinel datasets, which have 80 and 107 negative WSIs, respectively.

**Table 4:** Number of negative WSIs that had some FP areas introduced by the segmentation refinement.

Dataset	Method	# WSIs, softmax scores in ranges			
		0.55 to 0.95	0.65 to 0.95	0.75 to 0.95	0.85 to 0.95
Camelyon	Baseline	77	80	79	77
	SUA	7	6	9	25
Sentinel	Baseline	104	104	104	104
	SUA	31	31	42	65

There was no change in FROC-AUC or ROC-AUC observed from applying either of the FN detection methods compared with the original segmentation results.

## 7 Discussion

In this study, we aimed to determine whether spatially aggregating uncertainty could improve the ability to distinguish between TN and FN predictions in histopathology segmentation and bring clinical value by refining the segmentation heatmaps. First, we examined whether regions with incorrect negative predictions tended to have consistently different uncertainty values from the correct predictions. Our findings from experiment 1 indicate a significant correlation between the aggregated epistemic uncertainty over NPRs and FN predictions. Notably, we observed that broader NPRs, i.e., based on softmax scores in the 0.55 to 0.95, 0.65 to 0.95, and 0.75 to 0.95 ranges, yielded higher $F_{\beta =0.5}$ scores than narrower regions (with softmax scores in 0.85 to 0.95 range), suggesting that combining more pixels improved spatial uncertainty estimates. The baseline achieved substantially lower $F_{\beta =0.5}$ scores, confirming that it is advantageous to incorporate the spatial information within the uncertainty estimation. Given our definition of FN as containing at least 90% of tumor pixels, these results are highly promising, suggesting that spatial epistemic uncertainty could enhance the performance of a DL system for breast cancer metastases segmentation.

To evaluate the practical benefits of FN-detection-based heatmap refinement compared with utilizing the underlying uncertainty directly, we proposed the FNCR metric. The left plot in Fig. 6 indicates that the detected FN areas with the SUA framework enables a relatively successful refinement of in-domain segmentation heatmaps with most bootstrap runs achieving an FNCR of around 0.98 for NPRs with softmax ranges of 0.55 to 0.95 and 0.65 to 0.95. In all cases, the baseline exhibited a substantially worse performance measured in the median FNCR. Furthermore, the baseline also had a higher variability in its performance compared with the SUA framework, as indicated by the length of the whiskers of the box plots. We conclude that the SUA framework based on NPRs with softmax ranges of 0.55 to 0.95 and 0.65 to 0.95 performed best on the heatmap refinement task.

The lack of generalizability is a severe problem affecting DL for pathology applications; hence an important question is if the SUA framework can to some extent mitigate the observed negative effects on the performance from the domain shift. Based on relatively high $F_{\beta =0.5}$ scores in Table 2, it seems that there is a possibility of successfully detecting FNs in the Sentinel data. A drop in the median value of the FNCR is relatively small on the segmentation heatmap refinement task for the domain shift for the NPRs with softmax ranges of 0.55 to 0.95, 0.65 to 0.95, and 0.75 to 0.95. The improvement in the average Dice score is slightly lower on the domain shift data compared with the in-domain data (see Table 3). However, Table 4 reveals that a much higher number of negative WSIs had some incorrect refinement done on the Sentinel data compared with the Camelyon data. This is a worrying trend as increasing the number of false WSI predictions would severely compromise potential clinical benefits of uncertainty integration. We conclude that the utilized uncertainty estimation is sensitive to the domain shift and, hence, may not be suitable in domain shift scenarios. This is consistent with observations in previous studies.^14^^,^^41^ Even if uncertainty estimation can bring tangible value under in-domain data assumption, it is essential to have approaches that are able to handle the domain shift scenarios. As our results indicate, unfortunately, the SUA framework is unable to address this problem. Ensuring that uncertainty estimation is robust to domain shifts and hence helps to alleviate the drop in performance due to poor generalization of the DL algorithms is a very important future research topic.

It is challenging to evaluate what tangible benefits the incorporation of uncertainty with a DL model may provide. The average Dice score seems to be insensitive to some changes in prediction heatmaps. This may be caused by difference in the size of metastases, i.e., macro metastases are very large and, hence, more easily detectable by DL algorithms and pathologists. The average Dice score appears to primarily reflect the accurate segmentation of this type of metastases. This is supported by the minimal difference in the average Dice score between unmodified segmentation for the Camelyon and Sentinel datasets, whereas FROC-AUC indicates a significant difference in segmentation performance between these two datasets. The observation is consistent with prior work.^38^ It is difficult to say if the observed 0.003 Dice score improvement after refinement with the SUA framework could bring some clinical value because the decrease of 0.006 in the average Dice score on the original segmentation of the Sentinel data when comparing with the Camelyon data is also small.

Given the strong correlation between the SUA output and the regions with incorrect negative predictions observed in experiment 1, the uncertain clinical benefit brought by the uncertainty-based refinement in experiment 2 is a somewhat disappointing result, which highlights a potential need for improving the underlying uncertainty estimation approach. This is a surprising finding because several previous works reported promising performance of the DE uncertainty estimation method. However, their evaluation primarily focused on the correlation between incorrect predictions and the uncertainty values. Our study emphasizes the importance of evaluating the clinical value by acting upon the uncertainty information.

**Fig. 7:**
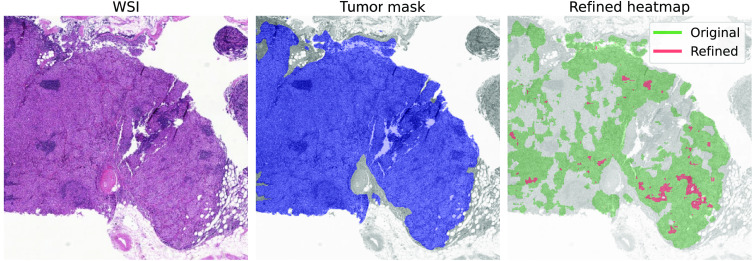
Example of a refined WSI from the Camelyon data via the SUA framework based on NPRs with the 0.55 to 0.95 softmax range. “Tumor mask” is the ground truth tumor annotation.

It seems that we are able to fill in some missed gaps in the segmentation (see Fig. 7), but ideally, we would aim to detect completely missed tumor metastases. However, defining what is a missed metastasis is not straightforward. For example, how far should a missed metastasis be from the correctly segmented one to have clinical relevance? If a patient has a macro metastasis, micro metastases become less important for the diagnostic decision, whereas isolated tumor cells usually do not have any impact.^42^ How to incorporate this knowledge in the evaluation of missed tumor metastases detection is an open question. Therefore, we believe that such a direction of work is not only very important but also requires careful considerations and experimentation. Hence, it could be the focus of future research.

A general challenge in computational pathology is to determine which evaluation metrics represent well the clinical impact of an approach. The Dice score does not take into consideration the prevalence of FP predictions, which can cause severe problems in clinical practice. To avoid data leakage, we are not able to reliably use FROC-AUC either. It would require tuning the segmentation threshold for each test data and using them in the heatmap refinement steps. We consider such a process to be clinically unrealistic. Utilizing thresholds learned on validation data resulted in no change in FROC-AUC and ROC-AUC after the refinement. Future work could aim to propose metrics that would more closely represent performance from a clinical point of view.

As mentioned, our approach is appealing because it can generalize to different combinations of DL architectures, targeted tasks in digital pathology, and uncertainty estimation techniques. The results indicate that the chosen base uncertainty estimation method is not able to significantly boost the DL performance on breast cancer metastasis segmentation in lymph nodes. However, several other studies have shown the potential benefits of uncertainty estimation in other DL applications in digital pathology.^27^^,^^43^ Hence, our observed improvement of utilizing SUA compared with uncertainty estimation directly on in-domain data indicates that the SUA approach could improve the obtained benefits from uncertainty estimation in the previously studied scenarios.

There are several limitations of this work. Only one uncertainty estimation method, i.e., the DE method, was tested with the SUA framework. Because the SUA framework is independent of the uncertainty estimation approach, investigating alternative uncertainty estimation techniques that are more suitable for computational pathology applications and that could be combined with the SUA framework could provide an unquestionable clinical impact. We believe that this direction could prove valuable. Moreover, in this study, we focused on the breast cancer metastasis segmentation task due to the clinical value of addressing this problem with DL assistance. It would be valuable to confirm that the observed benefits apply to other important segmentation tasks in digital pathology, such as nuclei segmentation.

## 8 Conclusion

Our analysis indicates that the SUA framework proposed for FN detection exhibits encouraging outcomes on in-domain data, with a noticeable correlation between FN regions and the aggregated uncertainty. Moreover, utilizing SUA for refinement of segmentation heatmaps further confirms its potential benefits compared with utilizing the underlying uncertainty directly. However, utilization of the chosen uncertainty estimation technique did not result in substantial improvements on the segmentation outcome. This negatively affected the effectiveness of segmentation refinement via SUA. We recommend future work to focus on determining a more robust uncertainty estimation method to combine with the SUA framework as well as further improving the evaluation approaches to quantify the potential benefits in realistic clinical settings.

## Data Availability

Only anonymous and publicly available data are used in this study; hence the data can be utilized in legal and ethical medical diagnostics research without the further requirement for ethical approval nor the consent from all subjects. The Camelyon dataset^36^ analyzed during the current study is available in the official GigaScience repository, http://gigadb.org/dataset/100439. The images from the Sentinel dataset used in this study are publicly available at the AIDA data hub, https://datahub.aida.scilifelab.se/10.23698/aida/brln. The annotations are not currently accessible to the public due to their use in an ongoing study. However, interested parties can obtain the dataset and the annotations from the corresponding author upon request. It is anticipated that they will be made publicly available once the study is completed. The code can be shared upon request, please contact Milda Pocevičiūtė at milda.poceviciute@liu.se.
